# Factors influencing residents' willingness to choose medical institutions first for treatment in the highland agricultural and pastoral areas of Qinghai province: a study based on the Anderson model

**DOI:** 10.3389/frhs.2026.1829554

**Published:** 2026-06-23

**Authors:** Yitao Ren, Shukui Han, Xin Ma, Yingchen Gao, Fangyun Hou, Tong Li, Ziyu Zhang, Panpan Song, Jinxiang Ma, Zhengyuan Xu, Bin Li, Hongru Chen

**Affiliations:** 1 Department of Public Health, Qinghai University Medical College, Xining, China; 2 Department of Medical Technology, Qinghai Institute of Health Sciences, Xining, China

**Keywords:** influencing factors, residents in highland agricultural and pastoral areas, Shapley value method, the Anderson model, willingness of residents' first choice of medical institutions

## Abstract

**Background:**

Previous studies on healthcare choice have rarely focused on highland agricultural and pastoral areas, where geographic barriers and unique sociocultural contexts may alter the determinants of medical institution preference. Moreover, most research has not quantified the relative contribution of different factors, limiting policy prioritization. This study fills these gaps by applying Anderson's behavioral model and Shapley value decomposition to identify and rank factors influencing first-choice medical institutions in Qinghai Province.

**Method:**

Data from 1,443 residents were analyzed using Anderson's behavioral model. Chi-square tests, binary logistic regression, and Shapley value decomposition were applied to quantify associations and relative contributions of predisposing, enabling, and need factors.

**Results:**

Overall, 62.7% preferred primary healthcare institutions. Multivariate analysis showed that older age (40–59 years: OR = 2.025, 95% CI: 1.383–2.967; ≥60 years: OR = 1.787, 95% CI: 1.122–2.855), having medical insurance (OR = 3.948, 95% CI: 2.303–6.938), and better self-rated health (moderate: OR = 1.471, 95% CI: 1.006–2.160; healthy: OR = 1.889, 95% CI: 1.291–2.776) increased the likelihood of choosing non-primary institutions. Conversely, longer travel distance (>15 min) strongly favored primary care (OR = 0.067, 95% CI: 0.046–0.096), as did higher education (junior college or above: OR = 0.285, 95% CI: 0.177–0.455) and having a chronic disease (OR = 0.698, 95% CI: 0.488–0.991). Shapley decomposition revealed that enabling factors contributed 66.55% of the model's explanatory power, with distance alone accounting for 59.01%; predisposing factors contributed 27.78% (education: 18.39%), and need factors contributed 5.68%.

**Conclusion:**

Geographic accessibility and enabling factors dominate healthcare choices in Qinghai's pastoral areas. Policy should prioritize reducing travel barriers, adjusting insurance incentives, and strengthening primary care capacity for chronic diseases.

## Introduction

Globally, rural populations face escalating health challenges due to limitations in living and working conditions, socioeconomic status, and access to healthcare services (1). This urban-rural disparity is most evident in the increasing incidence and mortality rates among rural residents. The primary risk factors contributing to health inequalities in these areas include geographic isolation, higher poverty levels, and a greater prevalence of health-risk behaviors (2). Since the onset of healthcare reforms in China in 2009, rural areas have been the focal point, with efforts directed at restructuring the healthcare system and improving the capacity and infrastructure of primary healthcare institutions, such as village clinics and township health centers (3, 4). A key component of these reforms is the introduction of the “15-min healthcare service circle,” aimed at ensuring that the majority of villagers can access basic healthcare services within a short distance, thus addressing critical health issues in rural regions (5). The conditions in pastoral areas closely resemble those in rural areas, yet they are more dispersed, which significantly hinders herders’ access to healthcare institutions. Furthermore, language barriers in the plateau agricultural and pastoral areas of Qinghai Province exacerbate the existing health disparities (6). While certain determinants of rural and pastoral health, such as geographic features and historical legacy, may be difficult to change, factors such as policy design, economic resource allocation, and healthcare delivery organization are amenable to improvement through targeted interventions (7, 8). Healthcare services, as essential interventions following the onset of disease, act as the final safety net for health maintenance. Ensuring equitable access to these services is essential for achieving health equity (9).

Disparities in socioeconomic and healthcare development between urban and rural areas in China have led to significant variations in the structure of healthcare service delivery across regions (10). The traditional healthcare model in China follows an inverted pyramid, with large hospitals at the apex, bearing the highest patient load, followed by medium-sized hospitals, and primary healthcare institutions positioned at the base (11). This patient flow pattern has contributed to escalating medical costs and stands in contrast to the internationally advocated model, where primary care serves as the gatekeeper of health (12).

Evidence suggests that effective public health strategies, when coordinated and implemented by primary healthcare institutions, are essential for reducing the incidence and mortality rates of chronic diseases (13). A robust primary healthcare system plays a crucial role in minimizing health disparities, controlling costs, and improving the quality of diagnostic, specialized, and inpatient services within communities (14). Establishing a tiered delivery system tailored to China's context could optimize healthcare-seeking behaviors, enhance the allocation of medical resources, strengthen the structure of health services, and promote vertical integration and collaboration across institutions (15, 16). Such a system can also reduce healthcare expenses for residents, ensure that patients receive care based on the severity of their conditions, and support the principle of treating minor illnesses at the community level while referring more complex cases to hospitals. This approach facilitates the orderly utilization of healthcare services, alleviates the burden on medical institutions, and enhances the equity and accessibility of healthcare. Numerous studies indicate that the choices of healthcare institutions among Chinese residents are influenced by factors such as socioeconomic status and service preferences (17–19). While some residents express a preference for primary healthcare institutions, many continue to favor municipal or district-level hospitals (20). These patterns highlight persistent challenges within China's healthcare system, including the underutilization of community health service centers and the continued demand for higher-level medical resources.

Qinghai Province, situated on the Tibetan Plateau, faces distinct challenges in its agricultural and pastoral regions, shaped by high altitudes, harsh climatic conditions, underdeveloped economic structures, and complex demographic profiles (21, 22). These contextual factors have collectively contributed to the long-standing underdevelopment of healthcare resources, particularly in terms of medical personnel, diagnostic equipment, and health infrastructure (23). Although substantial progress was made in poverty alleviation during the 13th Five-Year Plan period, pronounced disparities in the allocation of healthcare resources persist across regions (24). Primary healthcare institutions in highland agricultural and pastoral areas continue to exhibit limited service capacity, with some facilities lacking essential diagnostic tools (25, 26). Moreover, the severe shortage of qualified healthcare professionals has led many residents to bypass local services in favor of distant, higher-level hospitals (27). This phenomenon increases both the financial and temporal costs of care, further reinforcing existing urban-rural health disparities. In response, the government has implemented targeted interventions aimed at strengthening primary healthcare delivery, including infrastructure investment and the promotion of telemedicine (28). Nevertheless, systemic fragmentation and inefficiencies in healthcare resource allocation continue to impede the provision of integrated, high-quality healthcare services in rural Qinghai.

Overall, due to the unique geographic and socioeconomic conditions of Qinghai Province's highland agricultural and pastoral areas, residents demonstrate generally low utilization of healthcare services, a pattern similar to that observed in Tibet (29). Furthermore, there are notable disparities in the choice of medical institutions, which are closely tied to the distribution of healthcare resources, transportation accessibility, and traditional cultural beliefs. In recent years, China has implemented continuous reforms in its healthcare system, resulting in steady improvements in the primary healthcare network. These changes have led to shifts in residents’ healthcare-seeking behaviors and willingness to access services. However, Qinghai, with its vast territory, diverse minority populations, and unevenly distributed healthcare resources, continues to face challenges in providing adequate service capacity (30). This study investigates the preferred healthcare institutions among residents in plateau agricultural and pastoral areas, aiming to optimize resource allocation, enhance healthcare accessibility and quality in these areas, and provide a foundation for advancing the high-quality development of the healthcare system in Qinghai Province.

## Methods

### Data source and participants

This study employed a cross-sectional design. A multi-stage sampling strategy was used. First, we stratified by geographic region and ecological type (agricultural vs. pastoral) and selected three prefectures in Qinghai Province, namely Xining, Guoluo, and Huangnan (mean altitude >3,000 m). Second, within each stratum, we selected one to two counties or districts based on population proportions. Third, due to remote and dispersed settlement patterns, we recruited participants using convenience sampling at township health centers, village clinics, and community gatherings. A total of 1,443 eligible participants were enrolled voluntarily.

Inclusion criteria: (1) age ≥18 years; (2) local household registration and residence in the area for >6 months/year; (3) ability to communicate and provide informed consent; (4) willingness to participate. Exclusion: cognitive impairment preventing questionnaire completion.

The main outcome, defined as first choice of medical institution, was measured by a single question that asked “when you or a family member feels unwell, which institution you visit first.” Response options included village clinic, township health center, county hospital, city hospital or above, private clinic, and others. For analysis, this multi-category variable was dichotomized: first choice = 1 if the participant selected either village clinic or township health center (representing primary care), and first choice = 0 for all other options.

### Procedure

Standardized investigators obtained written informed consent from all participants prior to the survey (July–December 2024). A two-mode data collection strategy was used: face-to-face paper questionnaires in Xining, and online surveys via SoJump in Guoluo and Huangnan due to remote accessibility constraints. To minimise selection bias, a stratified convenience sampling design was adopted. In Xining, participants were recruited through convenience sampling at township health centers and community gatherings; in Guoluo and Huangnan, online links were distributed by community health workers and village leaders, with family assistance offered to residents lacking smartphone access or literacy. A total of 1,443 valid responses were obtained (response rate 97.6%). To assess potential mode-induced bias, we compared key variable distributions between the two subsamples and re-estimated the main logistic model using a Probit model; the direction, magnitude, and significance of coefficients remained virtually unchanged. This consistency suggests limited mode-related bias, although residual confounding from internet access and digital literacy cannot be ruled out.

### Ethics statement

The study was ethically approved by the Medical Research Ethics Committee of Qinghai University Medical College (Approval No. 2023–027) and conducted in accordance with the Declaration of Helsinki. Written informed consent was obtained from all participants prior to the questionnaire survey.

### Measurement

With the assistance of participants, a self-developed structured questionnaire was administered to address the research questions. Residents’ preferences for healthcare institutions (dichotomized as described above) were used as the dependent variable. Based on the literature and the availability of variables in existing databases, gender, age, ethnicity, marital status, education level, health insurance status, monthly income, chronic disease management, distance to the nearest healthcare institution, self-rated health status, depression, and frailty were included as covariates (31, 32). The questionnaire was developed in accordance with the research objectives, study design, relevant health policies, and a comprehensive review of the literature. Standardized scales were used to assess depression and frailty. The dichotomization followed a binary response format (yes = 1, no = 0) consistent with previous approaches (11).

### Depression

The Nine-item Patient Health Questionnaire (PHQ-9) (33) consists of nine items, each with four response options: “never” (0), “several days” (1), “more than half the days” (2), and “nearly every day” (3). The total score ranges from 0 to 27, with higher scores indicating more severe depressive symptoms. Based on established cutoff points, scores of 0–4 indicate no depression or clinically insignificant symptoms, 5–9 suggest mild depression, 10–14 indicate moderate depression, and 15–27 reflect severe depression. A score of 5 or above is considered positive, suggesting the presence of depressive symptoms. The internal consistency of the scale in this study was high, with a Cronbach's alpha coefficient of 0.841.

### Frailty

The FRAIL scale (34) consists of five components: fatigue, increased resistance or reduced endurance (difficulty ascending a single flight of stairs), decreased physical activity (difficulty walking one block independently), comorbidity (the presence of more than five diseases), and weight loss (a greater than 5% reduction in body weight over the past year). Each item is assigned a score of 1, yielding a maximum possible score of 5. A score of 0 denotes no frailty and indicates a robust older adult, scores between 1 and 3 suggest pre-frailty, and a score exceeding 3 indicates frailty. In this study, the Cronbach's alpha coefficient for the FRAIL scale was 0.759.

### Statistical analysis

A descriptive analysis was first conducted to summarize the frequencies (*N*) and percentages (%) of categorical variables, providing an overview of the sample distribution. Subsequently, cross-tabulations and Pearson's chi-square tests were employed to examine the associations between the Andersen model components—predisposing, enabling, and need factors—and residents’ willingness to select their initial healthcare institution (dichotomized). Multivariate logistic regression analyses were then performed, incorporating the three factor types in sequence, to estimate the associations between healthcare utilization choices and sociodemographic characteristics, and to identify potential determinants of healthcare institution selection in the plateau pastoral areas of Qinghai Province. All statistical analyses were conducted using IBM SPSS Statistics Version 28.0 for Windows, with two-sided tests and a significance level set at *p* < 0.05. Results are reported as odds ratios (ORs) with corresponding 95% confidence intervals (CIs). Cases with missing values were excluded from all analyses. Finally, a regression-based Shapley value decomposition method was applied to quantify the relative contributions of each dimension and significant variables, indicating the weight of each factor. This method allows for the assessment of both individual and grouped contributions of explanatory variables to the variance in the outcome. All statistically significant factors of binary logistic regression included in the study were entered into the model, and the command was executed in Stata. The resulting Shapley values and their corresponding percentages (contribution rates) were reported. Model robustness was evaluated using the Probit model as described in the Procedure section; no further Tobit or truncated regression analyses were performed.

## Results

### Sample Characteristics and Regression Analysis

A total of 1,443 valid questionnaires were collected, of which 43.31% were male. Among the participants, 25.32% were aged 60 or older, with an average age of 49.0 years. Ethnic minorities represented 79.42% of the sample, and 37.01% of participants were married. Furthermore, 63.55% had an education level at or below primary school. The majority of participants (92.10%) had medical insurance. Regarding economic status, 49.34% reported a monthly income below 1,000 RMB. Exactly 40.96% of participants could access the nearest medical facility within 15 min. The average depression score was low, with 59.25% showing no signs of depression. Notably, 47.47% of participants had at least one chronic disease, and 47.61% reported their health as poor. Only 42.13% of participants indicated they experienced no debilitating symptoms (Table 1).

**Table 1:** Choice of medical institutions with different sociodemographic characteristics (*n*, %).

Variables	Classification	Total	Primary health institutions	Non-primary health institutions	*χ* ^2^	*P*-value
Predisposing factors						
Gender	Male	625 (43.31)	426 (47.12)	199 (36.92)	14.319	<0.001
	Female	818 (56.69)	478 (52.88)	340 (63.08)		
Age	18–39	472 (32.71)	229 (22.67)	267 (49.53)	118.729	<0.001
	40–59	607 (42.07)	440 (48.67)	167 (30.98)		
	≥60	364 (25.23)	259 (28.65)	105 (19.48)		
Ethnicity	Han	297 (20.58)	197 (21.79)	100 (18.55)	88.039	<0.001
	Zang	532 (36.87)	253 (27.99)	279 (51.76)		
	Hui	579 (40.12)	431 (47.68)	148 (27.46)		
	Others	35 (2.43)	23 (2.54)	12 (2.23)		
Marital status	Single	865 (59.94)	590 (65.27)	275 (51.02)	29.012	<0.001
	Married	534 (37.01)	288 (31.86)	246 (45.64)		
	Separated/Divorced	24 (1.66)	14 (1.55)	10 (1.86)		
	Widowed	20 (1.39)	12 (1.33)	8 (1.48)		
Education level	Primary school or lower	917 (63.55)	664 (73.45)	253 (46.94)	193.954	<0.001
	Secondary school	232 (16.08)	150 (16.69)	82 (15.21)		
	High school	78 (5.41)	43 (4.76)	35 (6.49)		
	Junior College or above	216 (14.97)	47 (5.20)	169 (31.35)		
Enabling factors						
Insurance status	Yes	1,329 (92.10)	34 (3.76)	80 (14.84)	56.986	<0.001
	No	114 (7.90)	870 (96.24)	459 (85.16)		
Incoming monthly (RMB)	<1,000	712 (49.34)	476 (52.65)	236 (43.78)	71.646	<0.001
	1,001–3,000	455 (31.53)	258 (28.54)	197 (36.55)		
	3,001–5,000	179 (12.40)	107 (11.84)	72 (13.36)		
	>5,000	97 (6.72)	63 (6.97)	34 (6.31)		
	<15 min	591 (40.96)	546 (60.40)	45 (8.35)	379.293	<0.001
	≥15 min	532 (59.04)	358 (39.60)	494 (32.28)		
Depression	Normal	855 (59.25)	536 (59.29)	319 (59.18)	6.081	0.193
	Mild	380 (26.33)	225 (24.89)	155 (28.76)		
	Moderate	127 (8.80)	86 (9.51)	41 (7.61)		
	Moderately severe	55 (3.81)	37 (4.09)	18 (3.34)		
	Severe	26 (1.80)	20 (2.21)	6 (1.11)		
Need factors						
Having chronic disease	Yes	685 (47.47)	423 (46.79)	262 (48.61)	0.447	0.504
	No	758 (52.53)	481 (53.21)	277 (51.39)		
Self-evaluation general health status	Unhealthy	687 (47.61)	385 (42.59)	302 (56.03)	44.491	<0.001
	Moderate	355 (24.60)	214 (23.67)	141 (26.16)		
	Healthy	401 (27.79)	305 (33.74)	96 (17.81)		
Frailty	Non-frail	608 (42.13)	382 (42.26)	226 (41.93)	0.208	0.901
	Pre-frail	655(45.39)	412(45.58)	243(45.08)		
	Frail	180(12.47)	110(12.17)	70(12.99)		

Of the 1,443 participants, 904 (62.6%) indicated a preference for primary healthcare institutions as their first choice (Table 2). The results show that, among the predisposing factors, gender (*χ*^2^ = 14.319, *P* < 0.001), age (*χ*^2^ = 118.729, *P* < 0.001), ethnicity (*χ*^2^ = 88.039, *P* < 0.001), marital status (*χ*^2^ = 29.012, *P* < 0.001), and education level (*χ*^2^ = 193.954, *P* < 0.001) were significantly associated with healthcare choices. Among the enabling factors, insurance status (*χ*^2^ = 56.986, *P* < 0.001), monthly income (*χ*^2^ = 71.646, *P* < 0.001), and distance to the nearest medical institution (*χ*^2^ = 764.943, *P* < 0.001) were significantly associated with healthcare choices. Among the need factors, depression (*χ*^2^ = 6.081, *P* = 0.193) was not statistically significant, whereas self-evaluation of general health status (*χ*^2^ = 44.491, *P* < 0.001) was significantly related to healthcare choices.

**Table 2:** The results of a binary logistic regression analysis for factors influencing the choice of medical institutions among residents in the agricultural and pastoral areas.

Factors	Variables	Model 1			Model 2			Model 3		
		OR value	95% CI	*P* value	OR value	95% CI	*P* value	OR value	95% CI	*P* value
	Gender									
Predisposing factors	Male	REF	REF	REF	REF	REF	REF	REF	REF	REF
	Female	2.271	1.457–3.558	<0.001	1.269	0.950–1.697	0.074	0.781	0.583–1.046	0.098
	Age									
	18–39	REF	REF	REF	REF	REF	REF	REF	REF	REF
	40–59	0.728	0.568–0.931	0.012	1.909	1.321–2.760	<0.001	2.025	1.383–2.967	<0.001
	≥60	1.719	1.257–2.350	0.001	1.557	1.001–2.429	0.051	1.787	1.122–2.855	0.015
	Ethnicity									
	Han	REF	REF	REF	REF	REF	REF	REF	REF	REF
	Zang	0.810	0,553–1.185	0.278	0.710	0.452–1.115	0.137	0.774	0.484–1.237	0.283
	Hui	1.265	0.917–1.741	0.150	1.382	0.956–1.997	0.085	1.421	0.977–2.069	0.066
	Others	1.469	0.659–3.456	0.360	1.090	0.425–2.863	0.858	1.054	0.407–2.805	0.915
	Marital status									
	Single	REF	REF	REF	REF	REF	REF	REF	REF	REF
	Married	0.894	0.677–1.185	0.435	1.039	0.752–1.439	0.818	1.082	0.776–1.513	0.644
	Separated/Divorced	0.691	0.287–1.747	0.418	0.838	0.293–2.407	0.739	0.789	0.270–2.298	0.662
	Widowed	1.319	0.490–3.754	0.590	2.115	0.708–6.364	0.176	1.614	0.543–4.842	0.385
	Educational level									
	Primary school or lower	REF	REF	REF	REF	REF	REF	REF	REF	REF
	Secondary school	0.669	0.478–0.938	0.019	0.729	0.488–1.091	0.123	0.682	0.455–1.023	0.064
	High school	0.558	0.340–0.922	0.022	1.121	0.638–1.976	0.692	1.031	0.581–1.833	0.918
	Junior College or above	0.162	0.109–0.240	<0.001	0.287	0.179–0.457	<0.001	0.285	0.177–0.455	<0.001
	Insurance status									
	No				REF	REF	REF	REF	REF	REF
	Yes				3.841	2.255–6.702	<0.001	3.948	2.303–6.938	<0.001
	Incoming monthly (RMB)									
	<1,000				REF	REF	REF	REF	REF	REF
	1,001–3,000				0.829	0.601–1.142	0.250	0.843	0.607–1.171	0.308
	3,001–5,000				0.738	0.476–1.146	0.175	0.694	0.446–1.082	0.106
	>5,000				1.114	0.636–1.968	0.708	1.137	0.646–2.019	0.659
Enabling factors	Distance from residence to nearest medical institution									
	<15 min				REF	REF	REF	REF	REF	REF
	≥15 min				0.065	0.324–1.333	0.235	0.067	0.046–0.096	<0.001
	Depression									
	Normal				REF	REF	REF	REF	REF	REF
	Mild				0.719	0.514–1.004	0.053	0.811	0.571–1.148	0.238
	Moderate				0.552	0.331–0.923	0.023	0.730	0.425–1.261	0.256
	Moderately severe				0.652	0.324–1.333	0.235	0.939	0.452–1.978	0.868
	Severe				0.838	0.287–2.686	0.754	1.193	0.399–3.945	0.760
	Having chronic disease									
	No							REF	REF	REF
	Yes							0.698	0.488–0.991	0.046
Need Factors	Self-evaluation general health status									
	Unhealthy							REF	REF	REF
	Moderate							1.471	1.006–2.160	0.048
	Healthy							1.889	1.291–2.776	0.001
	Frailty									
	Non-frail							REF	REF	REF
	Pre-frail							0.734	0.520–1.033	0.077
	Frail							0.940	0.579–1.524	0.801
	Constant	2.271		<0.001	4.218		<0.001	3.676		0.001

Table 2 presents an analysis of the three dimensions within Anderson's health behavior model. The three binary logistic regression models developed in this study were all statistically significant. Model 1, which includes only predisposing factors, shows significant effects for gender, age, and education level. Model 2, which incorporates both predisposing and enabling factors, reveals that besides age and education level remaining significant, insurance status and depression also exert significant effects. Model 3 results, which further include all three dimensions, indicate significant relationships between the choice of medical institutions and age, education level, insurance status, distance to the nearest medical institution, chronic diseases, and self-rated general health status. The Hosmer–Lemeshow test for Model 3 yielded *χ*^2^ = 9.18 (df = 8, *P* = 0.331), indicating good calibration, and the McFadden pseudo *R*^2^ was 0.352, suggesting acceptable model fit. Specifically, respondents who are aged above 40 years, have a lower education level, have medical insurance, live within a 15-min travel time to the nearest medical institution, have no chronic diseases, and report poor self-rated health are more inclined to choose primary healthcare facilities.

### Shapley decomposition of determinants of medical choice

To quantify the contribution of each factor to residents’ choice of medical institutions, we conducted a Shapley value decomposition based on the binary logistic regression model. As shown in Table 3, the explanatory power of the three dimensions of Andersen's health behavior model varied substantially. The total contribution of predisposing factors was 27.78% (with education level alone accounting for 18.39%), enabling factors contributed 66.55% (driven primarily by distance from residence to the nearest medical institution at 59.01%), and need factors contributed 5.68%. These results confirm that enabling factors, particularly geographic accessibility, are the primary determinants of healthcare choices in the rural and pastoral areas of Qinghai Province. Among individual variables, distance from residence to the nearest medical institution had the highest contribution (59.01%), followed by education level (18.39%) and insurance status (6.97%).

**Table 3:** Decomposition of Shapley values in the choice of medical institutions among residents in the agricultural and pastoral areas.

Variable		Shapley value	Contribution (%)
Predisposing factors	Gender	0.005	1.57
	Age	0.020	5.65
	Ethnicity	0.004	1.27
	Marital status	0.003	0.90
	Education level	0.065	18.39
Enabling factors	Insurance status	0.025	6.97
	Incoming monthly (RMB)	0.001	0.40
	Distance from residence to nearest medical institution	0.207	59.01
	Depression	0.001	0.17
Need factors	Having chronic disease	0.003	0.79
	Self-evaluation general health status	0.015	4.36
	Frailty	0.002	0.53
	Total	0.352	100.00

### Robustness test

To test the robustness of the factors significantly impacting the choice of medical institutions, this study further employed Probit regression to analyze healthcare service utilization data among residents in the agricultural and pastoral areas of Qinghai Province and compared the results with those from binary logistic regression. As shown in Table 4, there is little variation between the Probit and binary logistic estimates in terms of the significance of each coefficient. It is clear that the results of the binary logistic model constructed based on Anderson's health behavior model are robust.

**Table 4:** Probit regression analysis for robustness check of the health service utilization index.

Variables		Probit stability test		
		OR value	95% CI	Significance
Gender				
	Male	REF	REF	REF
	Female	−0.139	−0.311–0.032	0.109
Age				
	18–39	REF	REF	REF
	40–59	0.425	0.202–0.648	<0.001
	≥60	0.331	0.059–0.605	0.017
Ethnicity				
	Han	REF	REF	REF
	Zang	−0.153	−0.426–0.121	0.275
	Hui	0.209	−0.012–0.429	0.063
	Others	0.053	−0.501–0.622	0.852
Marital status				
	Single	REF	REF	REF
	Married	0.008	−0.183–0.200	0.932
	Separated/Divorced	−0.229	−0.835–0.385	0.469
	Widowed	0.188	−0.437–0.824	0.569
Educational level				
	Primary school or lower	REF	REF	REF
	Secondary school	−0.220	−0.501–0.622	0.852
	High school	0.015	−0.326–0.357	0.931
	Junior College or above	−0.719	−0.991–−0.451	<0.001
Insurance status				
	No	REF	REF	REF
	Yes	0.793	0.484–1.110	<0.001
Incoming monthly (RMB)				
	<1,000	REF	REF	REF
	1,001–3,000	−0.115	−0.306–0.076	0.237
	3,001–5,000	−0.201	−0.455–0.054	0.127
	>5,000	0.090	−0.246–0.429	0.599
Distance from residence to nearest medical institution				
	<15 min	REF	REF	REF
	≥15 min	−1.552	−1.748−1.362	<0.001
Depression				
	Normal	REF	REF	REF
	Mild	−0.114	−0.316–0.087	0.266
	Moderate	−0.200	−0.517–0.119	0.218
	Moderately severe	−0.041	−0.479–0.404	0.853
	Severe	0.125	−0.525–0.819	0.709
Having chronic disease				
	No	REF	REF	REF
	Yes	−0.216	−0.424– −0.009	0.041
Self-evaluation general health status				
	Unhealthy	REF	REF	REF
	Moderate	0.243	0.020–0.469	0.033
	Healthy	0.365	0.142–0.590	0.001
Frailty				
	Non-frail	REF	REF	REF
	Pre-frail	−0.167	−0.365–0.030	0.099
	Frail	−0.015	−0.297–0.267	0.915
Constant		0.745	0.290–1.202	0.001

## Discussion

To our knowledge, this is the first study to analyze factors associated with residents’ first choice of medical institutions in highland agricultural and pastoral areas of Qinghai Province using Andersen's behavioral model. We examined demographic characteristics, socioeconomic status, and physical/mental health conditions, and identified differences in healthcare choices associated with predisposing, enabling, and need factors. Our findings may inform future policies to improve healthcare access in these regions.

This study employed Anderson's medical service utilization model as a framework to explore the commonalities and differences influencing residents’ first choice of medical institutions in the agricultural and pastoral areas of Qinghai Province. Unlike lowland or urban settings, Qinghai's plateau pastoral areas are characterized by sparse population, harsh climate, long distances between settlements, and limited secondary/tertiary care facilities concentrated in a few county seats or the provincial capital Xining. These unique contextual factors directly shape the interpretation of our results.

Gender showed a marginally significant association with healthcare choice (OR = 0.781 for females vs. males, *p* = 0.098), suggesting that female residents tended to choose primary healthcare facilities more often than males (35–37). This may reflect that women in these regions prioritize accessibility for routine care, though the effect was not statistically robust.

Age was strongly associated with healthcare choice. Compared with residents aged 18–39 years, those aged 40–59 years (OR = 2.025, *p* < 0.001) and those aged ≥60 years (OR = 1.787, *p* = 0.015) were significantly more likely to choose non-primary institutions like hospitals (38–41). In the context of plateau pastoral areas, middle-aged and older adults often present with advanced stages of chronic diseases due to long-term outdoor labor and limited prior access to preventive care. Their preference for non-primary institutions may reflect a perceived need for specialized diagnostics that village clinics cannot provide. Alternatively, younger adults may be more willing to use primary facilities for minor or acute issues due to convenience (42–44). Education level also influenced healthcare choices. Compared with primary school or lower education, having a junior college degree or above was associated with a markedly lower likelihood of choosing non-primary institutions (OR = 0.285, *p* < 0.001), i.e., higher education was linked to a preference for primary care (45, 46).This finding, which appears contrary to some previous studies, may be explained by the local context: better-educated residents in remote pastoral areas may have greater health literacy and thus more rationally choose accessible primary care for common conditions, or they may have higher trust in primary care services after recent investments in village clinics. Another plausible explanation is that higher education is often associated with stable employment and residency in county towns, where primary care facilities are better equipped. Conversely, less educated individuals living in remote pastoral areas might bypass primary care due to limited health awareness. However, our data do not allow us to directly test this hypothesis. Future qualitative research is needed to explore these underlying mechanisms. Secondary school education showed a borderline protective effect toward primary care (OR = 0.682, *p* = 0.064), while high school education had no significant effect.

Among enabling factors, having medical insurance was strongly associated with healthcare choice. Having insurance greatly increased the odds of choosing non-primary institutions (OR = 3.948, *p* < 0.001) (47, 48).This is likely because insured residents face lower out-of-pocket costs at higher-level hospitals, enabling them to bypass primary care. In Qinghai's highland areas, where insurance coverage is nearly universal, this financial protection may inadvertently encourage direct use of non-primary facilities, which runs counter to the intended gatekeeping role of primary care under China's tiered diagnosis and treatment policy. The current policy promotes “first contact in primary care,” but our finding suggests that insurance design has not effectively steered patients downward, especially in pastoral areas where hospital visits involve long travel.

Geographic distance was strongly associated with healthcare choices. Residents with a travel time >15 min to the nearest facility were far more likely to choose primary care than those within 15 min (OR = 0.067) (49). By pastoral standards, a 15-min cutoff is extremely short—many herders live hours away. That such a pronounced effect appears at this low threshold underscores distance as the dominant factor. This finding challenges the feasibility of tiered care policy in remote areas: residents default to primary care out of geographic necessity, not because of a well-functioning referral system.

In this study, having a chronic disease was associated with a greater likelihood of choosing primary care (OR = 0.698, *p* = 0.046) (50, 51). This aligns with the expectation that chronic conditions require frequent follow-up, medication refills, and long-term management, making convenient and accessible primary care a practical choice (52, 53). Therefore, the observed preference may reflect an unmet need rather than satisfaction. Future research should explore whether chronic disease patients who choose primary care actually receive continuous medication supply and adequate monitoring.

Self-rated health status was significantly associated with healthcare choices. Compared with “unhealthy” respondents, those reporting “moderate” (OR = 1.471, *p* = 0.048) or “healthy” (OR = 1.889, *p* = 0.001) status were more likely to choose non-primary institutions (32, 54). Conversely, poor self-rated health favored primary care. A plausible explanation is that individuals with poor self-rated health may view their conditions as routine or manageable, preferring the convenience of primary care, or may have mobility limitations that hinder travel to distant hospitals (55, 56). By contrast, healthier individuals may proactively seek non-primary facilities for preventive services or have greater mobility and financial resources. This finding suggests that health education should highlight the value of primary care for preventive services even among healthy populations.

These findings suggest that in highland agricultural and pastoral areas of Qinghai Province, the choice of primary vs. non-primary institutions is shaped by distinct factors. Older age, having medical insurance, and better self-rated health drive residents toward non-primary facilities, whereas longer travel distance, higher education (junior college or above), chronic disease, and poor self-rated health favor primary care. Notably, the extremely low OR for distance (0.067) underscores that geographic barriers remain the strongest determinant of healthcare choices. To promote the appropriate use of primary care and achieve an effective tiered healthcare system, policymakers should consider: (1) improving transportation infrastructure and increasing primary care outreach (e.g., mobile clinics) in remote areas; (2) strengthening chronic disease management capacity at primary facilities; (3) adjusting insurance incentives to encourage first-contact primary care without increasing financial burden; and (4) enhancing public awareness of primary care capabilities, especially among younger, healthier, and insured populations who currently favor non-primary institutions.

## Conclusion

This cross-sectional study identifies factors associated with healthcare choices in rural and pastoral Qinghai, including geographic distance, age, insurance, education, chronic disease, and self-rated health. To strengthen primary care, the government could consider improving geographic accessibility, reforming insurance incentives, enhancing chronic disease management, and promoting public awareness of primary care capabilities. These actions may help reduce urban–rural and plateau–plain healthcare disparities. Strengthening coordination between primary and tertiary institutions is also crucial.

## Implications of the findings for future studies

First, to overcome the inherent limitation of the cross-sectional design, future research must employ longitudinal or cohort designs to establish causal relationships between enabling factors and primary care choice, particularly following policy or infrastructure changes in plateau pastoral areas. Second, localized qualitative studies are required to explain why ethnicity and gender were non-significant in our multivariate analysis. Such studies should explicitly account for regional characteristics: seasonal herding migration, extreme weather constraints, and the uneven distribution of healthcare facilities. Third, subsequent models must incorporate previously omitted variables—namely service capacity of primary institutions, residents’ trust in grassroots providers, ethnic medical customs, and the implementation of two-way referral policies—using validated instruments within multi-level analytical frameworks. This will reduce omitted variable bias and enhance model explanatory power.

Furthermore, intervention and implementation science research is necessary to develop and test culturally tailored health education programs, chronic disease management protocols, and mental health training for village doctors. Formal integration of inter-sectoral collaboration—linking health, social security, transportation, and education—is essential to address systemic resource fragmentation rather than relying on isolated policy adjustments.

## Limitations

This study has several limitations. First, the cross-sectional design precludes causal inference; only associations can be reported, and extrapolation requires caution. Future longitudinal studies such as cohort tracking of policy changes are needed to establish causality. Second, the mixed survey mode using face-to-face in Xining vs. online in Guoluo and Huangnan may introduce selection bias by excluding residents without internet access or low literacy. Although comparisons of key variables between subsamples and a Probit robustness check showed consistent results suggesting limited mode-induced bias, residual confounding such as digital literacy cannot be completely ruled out, and more rigorous adjustment was not performed. Third, several theoretically important variables were not collected, including service capacity of primary healthcare institutions, residents’ trust in grassroots medical services, ethnic medical customs, and implementation of two-way referral policies. This omission is primarily due to questionnaire length constraints and the difficulty of measuring these constructs in remote highland settings. Their absence may lead to omitted variable bias and reduce the model's explanatory power. For instance, trust in primary care might correlate with both education level and preference for primary institutions, potentially affecting our estimates. Fourth, self-reported data may incur recall bias. Despite these limitations, this study provides a critical foundation for improving healthcare utilization in plateau pastoral areas.

## Data Availability

The raw data supporting the conclusions of this article will be made available by the authors, without undue reservation.
