# Supplementary material for: Factors influencing residents' willingness to choose medical institutions first for treatment in the highland agricultural and pastoral areas of Qinghai province: a study based on the Anderson model
Source: Front Health Serv. 2026 Jun 23;6:1829554. doi: 10.3389/frhs.2026.1829554 (PMC13337931; doi:10.3389/frhs.2026.1829554)
Supplement: Supplementary file 1 [file Table1.docx]

**Questionnaire on the Willingness of Residents’ First Choice of Medical Institutions for Treatment in the Highland Agricultural and Pastoral Areas of Qinghai Province.**

Dear Healthcare Colleagues,

Hello. This survey is endorsed by the Health Commission of Qinghai Province and aims to improve the use of healthcare services among residents in high-altitude rural and pastoral areas. The reliability of the results depends on your thoughtful and objective responses. Please read each question carefully and answer based on your true experiences and feelings. This is an anonymous survey, and there are no right or wrong answers. We assure you that all information collected will be kept strictly confidential. Thank you for your support and cooperation.

**Table 1** **Demographic and characteristics of residents**(Please **√** under the serial number that matches your situation)

| 1 | Sex: (1) Male (2) Female | 2 | Age: |
| --- | --- | --- | --- |
| 3 | Your current marital status is: (1) Single (2) Married (3)Widowed (4)Divorced | | |
| 4 | Your ethnicity is: (1) Han (2) Zang (3) Hui (4) others | | |
| 5 | Your highest education is: (1) High school education or below (2) Technical secondary school (3) Junior College or above | | |
| 6 | Do you have health insurance?:(1) Yes (2) No | | |
| 7 | How long does it take you to get to the nearest healthcare facility? (1) <15 minutes (2) ≥15 minutes | | |
| 8 | Your monthly income (yuan) is: (1) <3000 (2) 3000~4999 (3) 5000~7999 (4) 8000~9999 (5) >10000 | | |
| 9 | Do you have a chronic diseases? (1) Yes (2) No | | |
| 10 | How would you assess your own health? | | |

**Table 2** **Social frailty** (Please **√** on the option that best suits your situation, depending on your actual feelings)

| **project** | | **Yes** | **No** |
| --- | --- | --- | --- |
| 1 | Do you have the ability to assist friends/family within the past year? |  |  |
| 2 | Did you participatie in any social/recreational activities during the past year? |  |  |
| 3 | Did you have feelings of loneliness in the preceding week? |  |  |
| 4 | Has your income over the past year been sufficient to cover your living expenses for the entire year? |  |  |
| 5 | Did you have someone to talk to every day? |  |  |

**Table 3 Depression** (Please √ based on your true feelings at work on the appropriate option that best reflects how often you feel)

| **Project** | | Never | Sometimes | often | Always |
| --- | --- | --- | --- | --- | --- |
| 1 | I often feels uninterested or bored with activities. |  |  |  |  |
| 2 | I Frequently experiences feelings of sadness, hopelessness, or depression. |  |  |  |  |
| 3 | I often struggles with falling asleep, staying asleep, or wakes up in the middle of the night; or conversely, sleeps excessively or experiences daytime sleepiness. |  |  |  |  |
| 4 | I often feels fatigued or lacks energy. |  |  |  |  |
| 5 | I Frequently experiences a lack of appetite or overeats. |  |  |  |  |
| 6 | I often feels dissatisfied with oneself—feels like a failure or has disappointed family members. |  |  |  |  |
| 7 | I often finds it difficult to concentrate, even when reading the newspaper or watching TV. |  |  |  |  |
| 8 | I often moves or speaks slowly to the point of drawing attention; or conversely, feels restless, pacing around, and easily irritable. |  |  |  |  |
| 9 | I often has thoughts of wishing to die or considering self-harm in some form. |  |  |  |  |
